# The associations between body and knee height measurements and knee joint structure in an asymptomatic cohort

**DOI:** 10.1186/1471-2474-13-19

**Published:** 2012-02-15

**Authors:** Andrew J Teichtahl, Anita E Wluka, Boyd J Strauss, Yuanyuan Wang, Patricia Berry, Miranda Davies-Tuck, Flavia M Cicuttini

**Affiliations:** 1 Department of Epidemiology and Preventive Medicine, School of Public Health & Preventive Medicine, Monash University, Alfred Hospital, Melbourne, Vic 3004, Australia; 2 Baker Heart Research Institute, Commercial Road, Melbourne, Vic 3004, Australia; 3 Department of Medicine, and Nutrition & Dietetics, Southern Clinical School, Monash University, Clayton, VIC, Australia; 4 Body Composition Laboratory, Clinical Nutrition and Metabolism Unit, Monash Medical Centre, Clayton, VIC, Australia; 5 Department of Epidemiology and Preventive Medicine, School of Public Health & Preventive Medicine, Monash University, Alfred Hospital, Melbourne, VIC 3004, Australia

**Keywords:** Knee height, Knee, Cartilage, Osteoarthritis

## Abstract

**Background:**

It has been suggested that knee height is a determinant of knee joint load. Nonetheless, no study has directly examined the relationship between anthropometric measures of height and knee joint structures, such as cartilage.

**Methods:**

89 asymptomatic community-based adults aged 25-62 with no diagnosed history of knee arthropathy were recruited. Anthropometric data (knee height and body height) were obtained by standard protocol, while tibial cartilage volume and defects, as well as bone area were determined from magnetic resonance imaging. Static knee alignment was measured from the joint radiograph.

**Results:**

All anthropometric height measures were associated with increasing compartmental tibial bone area (*p *≤ 0.05). Although knee height was associated with tibial cartilage volume (e.g. β = 27 mm^3 ^95% CI 7- 48; *p *= 0.009 for the medial compartment), these relationship no longer remained significant when knee height as a percentage of body height was analysed. Knee height as a percentage of body height was associated with a reduced risk of medial tibial cartilage defects (odds ratio 0.6; 95% confidence interval 0.4 - 1.0; *p *= 0.05).

**Conclusion:**

The association between increased anthropometric height measures and increased tibial bone area may reflect inherently larger bony structures. However the beneficial associations demonstrated with cartilage morphology suggest that an increased knee height may confer a beneficial biomechanical environment to the chondrocyte of asymptomatic adults.

## Background

A novel and interesting measurement that may be related to knee joint structure is knee height. Although yet to be formally examined, the rationale for knee height being an important determinant of joint structure is based on the hypothesis that greater limb length imparts larger moments around the knee, producing more torque and subsequent joint loads.

In the only study to have examined knee height, Hunter and colleagues (2005) demonstrated an increased prevalence of radiographic knee osteoarthritis (OA) in elderly Beijing residents with increased knee height [1]. This study comprised a population of Chinese aged 60 years or older and used radiographic disease as one of the measured end-points. Radiographic knee OA is heavily reliant on the osteophyte to classify disease. However OA is a disease of the whole joint and it is now well recognized that factors such as physical activity may be associated with the development of osteophytes, due to traction effects, while also being associated with beneficial effects on knee cartilage [2-5]. Thus, whether knee height is actually deleterious to individual joint structures is unclear. With the advent of magnetic resonance imaging (MRI), it is now possible to directly examine articular structures, such as cartilage, and to help better characterize the association between anthropometric measures of height and knee joint morphology. Moreover, MRI enables asymptomatic subjects, free from radiographic OA, who may however have very early degenerative changes, such as cartilage defects, to be examined in an attempt to help better understand the pathogenesis of pre-clinical knee OA.

The aim of this study was to examine whether body height, knee height and knee height as a percentage of body height are associated with knee joint structures in an asymptomatic community-based population.

## Methods

### Subjects

Eighty-nine community-based Caucasian Australian participants were recruited through local media and public/private/community weight loss clinics to take part in a community-based study of lifestyle factors on knee health. These 89 subjects were a subgroup of a larger cohort examining obesity and knee structure who had an X-ray of the knee available for analysis. Exclusion criteria included physician diagnosed arthritis, prior surgical intervention to the knee, previous significant knee injury requiring non weight bearing therapy, knee pain precluding weight-bearing activity for >24 h or prescribed analgesia, malignancy, inability to complete the study or contraindication to MRI.

The study was approved by Alfred Human Research and Ethics Committee and the Monash Standing Research Ethics Committee.

### Anthropometric data

Body weight was measured to the nearest 0.1 kg (shoes, socks, and bulky clothing removed) using a single pair of electronic scales. Body height was measured to the nearest 0.1 cm (shoes and socks removed) using a stadiometer.

**Figure 1:**
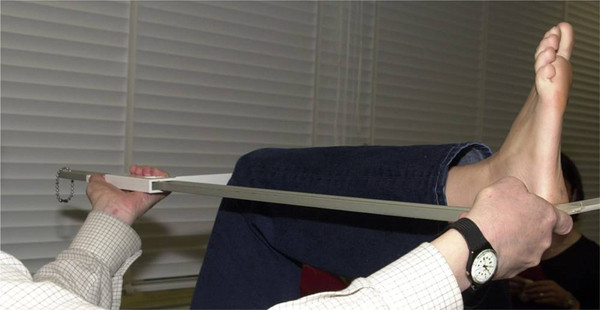
**Knee height measurement, Knee height was defined as the distance from the sole of the foot to the most anterior surface of the femoral condyles of the thigh (medial being more anterior), with the ankle and knee each flexed to a 90° angle**.

Knee height was measured by a single trained observer using the standardized procedures as described by Zhang [6]. Knee height was defined as the distance from the sole of the foot to the anterior surface of the femoral condyle of the thigh, with the ankle and knee each flexed to a 90° angle (see Figure 1). It was measured on each of the participants while supine on the examination table using a sliding caliper on the leg that had undergone MRI to the nearest 0.1cm. The coefficient of variation (CV) for the measure of knee height was 3.9%. Knee height as a percentage of totally body height was then calculated by dividing the knee height (cm) by the body height (cm), and multiplying by 100.

### Magnetic resonance imaging (MRI)

An MRI of the dominant knee of each subject (defined as the lower-limb from which the subject leads from when initiating gait) was performed. Knees were imaged in the sagittal plane on a 1.5-T whole body magnetic resonance unit (Philips, Medical Systems, Eindhoven, the Netherlands) using a commercial transmit-receive extremity coil. The following sequence and parameters were used: T1-weighted fat saturation 3D gradient recall acquisition in the steady state (58ms/12ms/55°, repetition time/echo time/flip angle) with a 16 cm field of view, 60 partitions, 512 × 512 matrix and acquisition time 11 min 56 s (one acquisition). Sagittal images were obtained at a partition thickness of 1.5 mm and an in-plane resolution of 0.31 × 0.31 mm (512 × 512 pixels) [7,8].

Cartilage volumes and bone areas at the tibia were determined by image processing on an independent workstation using the Osiris software (University Hospital of Geneva, Geneva, Switzerland). Contours were drawn around the tibia in images 1.5mm apart on sagittal views via disarticulation contours around the cartilage boundary. Measurements were performed by one trained observer with independent random cross checks blindly performed by a second trained observer, both blinded to subject identification, and data collected. The CVs for tibial cartilage volume and bone area were both 2.1% [7,9].

Cartilage defects were graded on the MR images with a classification system that has been previously described [8,10]: grade 0 = normal cartilage; grade 1 = focal blistering and intracartilaginous low-signal intensity area with an intact surface and bottom; grade 2 = irregularities on the surface or bottom and loss of thickness of less than 50%; grade 3 = deep ulceration with loss of thickness of more than 50%; grade 4 = full-thickness cartilage wear with exposure of subchondral bone. Medial and lateral defects were graded for the tibial compartment. The presence of a cartilage defect was defined by grade 1 or higher. Intraobserver reliability assessed in 50 MR images (expressed as intraclass correlation coefficient) was 0.90 for the medial compartment and 0.89 for the lateral compartment [11].

Osteophytes were measured from MR images, which have been shown to be more sensitive than X-rays [12]. Osteophytes were measured from coronal images by two independent trained observers. In the event of disagreement between observers, a third independent observer reviewed the MRI. Intra-observer and inter-observer reproducibility for agreement on osteophytes ranged between 0.85 and 0.93 (κ statistic).

### Knee alignment

Knee angles were measured by a blinded observer from standing anterior-posterior radiographs using the software program Osiris, as previously described [13]. Lines were drawn through the middle of the femoral shaft and through the middle of the tibial shaft. The angle subtended at the point at which these two lines met in the centre of the tibial spines, and was recently validated by Hinman et al [13] as an alternative to the mechanical axis on full-leg radiographs. Knee angles were considered as a continuum ranging from 0 to 360°, with 0° representing extreme varus and 360° representing extreme valgus. Although these degrees of varus and valgus are not clinically observed, a continuous range was used to avoid defining varus and valgus from an arbitrarily chosen midline value. Intra-observer reliability (expressed as ICC) was 0.98 [14].

### Statistical Analysis

Cartilage volume and bone area was initially assessed for normality (i.e. conformed to a bell-shaped distribution and Wilk-Shapiro test) before being regressed against knee height variables using a linear regression model. The presence of tibial cartilage defects were categorised as a dichotomous outcome (yes/no), therefore logistic regression was used. Confounders including age, weight and gender were adjusted for in the regression models. We have also adjusted analyses for static knee angle, in an attempt to control for any influence of knee malalignment. Since cartilage volume is a known determinant of cartilage defects, defect analyses have subsequently been adjusted for cartilage volume. Likewise, metaphyseal bone area is a known determinant of cartilage volume, and as such, has been adjusted for in the appropriate analyses. Finally, all analyses were adjusted for the presence of MRI determined osteophytes based on previous work [1]. A P value of less than 0.05 was considered to be statistically significant. All analyses were performed using the SPSS statistical package (standard version 16.0, SPSS, Chicago, IL).

## Results

Subject characteristics are shown in Table 1. 89 adults (82% female) aged between 25 and 62 years (mean age 47.4 years) participated in the study. The average height was 167.6cm (SD ± 9.1cm), while the average knee height and knee height as a percentage of body height was 51.1cm (SD ± 3.6cm) and 30.5% (SD ± 1.2%) respectively.

**Table 1:** Mean subject demographics, knee height measures and MRI variables

	N = 89
**Age (years)**	47.4 (8.6) [28-62]
**Gender--Number of females (% females of sample size)**	73 (82)
**Height (cm)**	167.6 (9.1) [152-194]
**Weight (kg)**	90.2 (26.1) [49-164]
**BMI (kg m^-2^)**	32.2 (9.2) [16.9-51.4]
**Knee height (cm)**	51.1 (3.6) [42.8-62.7]
**Knee height as a percentage of body height (%)**	30.5 (1.2) [26.4-33.9]
**Medial tibial cartilage volume (mm^3^)**	1020 (284) [564-1936]
**Lateral tibial cartilage volume (mm^3^)**	1329 (394) [750-2816]
**Medial tibial bone area (mm^2^)**	1893 (232) [1337-2574]
**Lateral tibial bone area (mm^2^)**	1458 (220) [1088-2120]
**Presence of medial tibial cartilage defects (%)**	47 (53)
**Presence of lateral tibial cartilage defects (%)**	79 (89)
**Static knee angle (degrees)**	181 (3) [173-191]
**Presence of MRI osteophytes--Yes (% of sample size)**	31 (35)

Results reported as mean (± standard deviation) unless otherwise stated

Results in brackets [x] represent range of continuous variables

The association between height measurements and knee joint structures are shown in Table 2. Body height was associated with tibial bone area and cartilage volume (β ranging from 6-11 mm^2 ^for bone area results; and 10-12 mm^3 ^for cartilage volume results; all *p *≤ 0.06). Knee height was associated with medial (β = 27 mm^3 ^95% CI 7- 48; *p *= 0.009) and lateral (β = 42 mm^3 ^95% CI 19- 65; *p *= 0.001) tibial cartilage volume after adjustment for confounders. That is, medial cartilage volume was increased by 27 mm^3 ^for every 1 cm increase in knee height, while lateral cartilage volume was increased by 42 mm^3 ^for every 1cm increase in knee height. In relative terms, for the medial compartment, this data demonstrated a 2.6% increase in the average total cartilage volume, for every 1cm knee in knee height. However, when knee height as a percentage of body height and tibial cartilage volumes were examined, the medial tibial cartilage volume was no longer significantly associated (*p *= 0.63), and the lateral tibial cartilage volume now only tending toward significance (*p *= 0.11). Knee height as a percentage of body height was associated with bone area (β ranging from 43 - 50 mm^2^; all *p *≤ 0.02). Although knee height was not associated with the risk of cartilage defects, knee height as a percentage of body height was associated with a reduced risk of the presence of medial tibial cartilage defects (Odds ratio 0.6; 95% Confidence interval 0.4 - 1.0; *p *= 0.05). Both body height and knee height tended to be associated with an increased risk for the presence of MRI determined osteophytes (β = 1.2, 95% CI 1.0 - 1.5; *p *≤ 0.06).

**Table 2:** The association between anthropometric height measures and tibial cartilage volume, defects and bone area

	Body height				Knee height				Knee height as percentage of body height			
	
	Univariate	*P*	Multivariate	*P*	Univariate	*P*	Multivariate	*P*	Univariate	*P*	Multivariate	*P*
Bone area^1^												
*Medial*	16 (11, 20)	<0.001	11 (4, 17)	0.001	44 (34, 54)	<0.001	37(23 52)	0.001	72 (32, 111)	0.001	50 (14, 87)	0.007
*Lateral*	12 (8, 17)	<0.001	6 (0, 12)	0.06	36 (25, 47)	<0.001	27 (11, 42)	0.001	65 (28, 103)	0.001	43 (7, 79)	0.002

Cartilage volume^1^												
*Medial*	19 (14, 24)	<0.001	10 (4, 17)	0.001	51 (38, 64)	<0.001	27 (7, 48)	0.009	71 (21, 120)	0.006	11 (-33, 54)	0.63
*Lateral*	28 (21, 35)	<0.001	12 (3, 21)	0.009	79 (63, 93)	<0.001	42 (19, 65)	0.001	129 (62, 195)	<0.001	44 (-10, 98)	0.11

Cartilage defects^2^												
*Medial*	1.0 (0.9, 1.1)	0.31	1.0 (0.9, 1.1)	0.62	1.0 (0.9, 1.1)	0.97	0.9 (0.7, 1.1)	0.29	0.8 (0.5, 1.1)	0.17	0.6 (0.4, 1.0)	0.05
*Lateral*	1.0 (0.9, 1.1)	0.99	1.0 (0.9, 1.1)	0.86	1.0 (0.8, 1.2)	0.84	0.9 (0.7, 1.3)	0.59	1.1 (0.6, 1.9)	0.74	0.8 (0.4, 1.7)	0.55
MRI osteophytes^2^	1 (0.9, 1.1)	0.63	1.1 (1.0, 1.2)	0.01	1.0 (0.9, 1.2)	0.53	1.2 (1.0, 1.5)	0.06	1.1 (0.8, 1.6)	0.58	1.0 (0.6, 1.5)	0.94

Bone area (mm^2^) adjusted for age, gender, weight, static knee angle and presence of tibiofemoral osteophytes (yes/no)

Cartilage volume (mm^3^) adjusted for age, gender, weight, respective bone area, static knee angle and presence of tibiofemoral osteophytes (yes/no)

Cartilage defects adjusted for age, gender, weight, respective cartilage volume, static knee angle and presence of tibiofemoral osteophytes (yes/no)

MRI osteophytes adjusted for age, gender and BMI.

**^1^**Results expressed as **beta **coefficient of regression (95% confidence interval)

^2^Results expressed as odds ratio (95% confidence interval)

## Discussion

In asymptomatic community-based adults, we have demonstrated that increased anthropometric height measures are associated with increased tibial bone area, while knee height as a percentage of body height is associated with a reduced risk of medial tibial cartilage defects. We have also found that knee height is associated with knee cartilage volume. The associations between increased anthropometric measures and increased bone area may simply reflect inherently larger bony structures. However the beneficial associations demonstrated with cartilage morphology suggest that an increased knee height may confer a beneficial biomechanical environment to the chondrocyte of asymptomatic adults.

In the only previous study to have examined knee height, radiographic knee OA was associated with increased knee height among Beijing residents aged 60 years or older [1]. According to the Kellgren-Lawrence grading system, the diagnosis of radiographic OA is heavily reliant on the presence of osteophytes to classify disease. The role of osteophytes in disease pathogenesis remains unclear, but much of the previous study's association between knee height and radiographic OA [1] may have been mediated by the presence of osteophytes. To further explore this concept, we found that knee height tended to be significantly associated with an increased risk for the presence of MRI osteophytes (OR 1.2; 95% CI 1.0 - 1.5; *p *= 0.06). MRI has been shown to be more sensitive than the joint radiograph at determining the presence of osteophytes [12]. Furthermore, we demonstrated that increased knee height is associated with increased tibial bone size, which supports knee height being inherently linked to bone morphology and may not necessarily be detrimental to other structures, such as cartilage. This leads to the quandary of determining whether increased knee height is to the detriment of the knee joint. In this study, we substantiate Hunter et al. results by demonstrating a possible deleterious association, particularly with the osteophyte [1], a key criterion for the diagnosis of radiographic OA.

Nonetheless, despite the deleterious association with the knee osteophyte, we have demonstrated that an increased knee height is associated with increased knee cartilage volume, although this relationship did not persist when knee height as a percentage of body height was examined. This indicates that although knee height is associated with cartilage volume, the relationship is attenuated when the overall stature of an individual is accounted for. In contrast, knee height as a percentage of body height (but not isolated knee height) was associated with a reduced risk of medial tibial, but not lateral cartilage defects. Why isolated knee height is associated with cartilage volume, and knee height as a percentage of body height is associated with reduced medial cartilage defects, but not vice versa, is unclear. However, the direction of these results is consistent and infer that increased knee height (either isolated, or relative to total body height) is associated with beneficial cartilaginous properties (increased cartilage volume and reduced cartilage defects) at the knee, suggesting a protective biological effect. Moreover, the medial compartment specific association between knee height as a percentage of body height and cartilage defects further substantiates an underlying biomechanical mechanism, since knee joint loads are predominantly directed medially [15]. Cartilage defects are surface lesions which, independent of cartilage volume, predict cartilage loss and pain in both people with and without knee OA [16-21]. Why increased joint load, which is speculated to result from an increase in knee height, either alone or as a percentage of body height, benefits the cartilage of asymptomatic people is unclear. It may be that healthy articular cartilage relies upon a certain degree of mechanical stimulation. In childhood, cartilage accrual is greater in physically active children [22], while in adults, forced immobility results in a rapid decline in knee cartilage [23,24]. Mechanical stimulation may therefore be imperative in maintaining cartilage health, although mechanocellular mechanisms may be easily perturbed when disease processes are activated by other means (e.g. in the setting of obesity).

There are a number of other factors that may also account for the differences between the results of our MRI study and the previous radiographic study. In contrast to our population which was Caucasian, the previous study examined Beijing residents. The Chinese have been demonstrated to have more valgus alignment of the distal femur than Caucasians [25]. Biomechanically, changes in varus-valgus alignment of the lower-limb can ameliorate the external knee adductor moment, which is the major determinant of joint load distribution at the knee [15]. In contrast to the previous study, we have adjusted for knee alignment. Moreover, we have examined a cohort of people without established knee OA. The disease status of a joint health may be a key determinant of how the chondrocyte responds to external loads. Similarly, articular structures may respond differently to the same stimuli over the life-span. Whereas the previous study of Beijing residents examined people aged 60 or over [1], we have examined a younger population with only 2.9% being aged 60 or over. In addition, we also examined knee height as a proportion of total body height, which may explain further differences in the findings.

This study has several limitations. Although we excluded people with a diagnosed arthropathy, we have not adjusted for the possibility of radiographic OA, despite adjusting for osteophytes. Since we did not have knee radiographs we determined the presence of osteophytes from MRI. This has previously been shown to be a more sensitive method for determining the presence of osteophytes than radiography [26]. Moreover, our main findings related to cartilage volume and defects were independent of bone area and cartilage volume respectively, both of which are strongly associated with radiographic knee OA [27,28]. Directly assessing cartilage volume is more sensitive than radiography for detecting early OA, since more than 10% of cartilage is already lost before any radiographic OA is detected [27]. Moreover, since our relatively small (n = 89) cohort was predominantly female (82%), recruited in part, from weight-loss clinics (mean BMI 32 kg m^-2^), we were limited in our ability to perform subgroup analyses based on gender. Additionally, we could not meaningfully analyse other subgroups, such as obesity classes, which impacts the generalisability of our findings. Nonetheless, we have limited the confounding effect of gender and weight, by adjusting for these in multivariate analyses. Finally, as this is a cross-sectional study, longitudinal data is needed to confirm the protective effect of an increased knee height on cartilage in those without knee OA.

## Conclusions

This is the first study to directly examine articular structures and anthropometrical measurements of height. We have found that in asymptomatic community-based adults, increased bone area is associated with increased measures of knee height. We also found that increased knee height measurements were associated with increased knee cartilage volume and a reduced risk for medial knee cartilage defects. The associations with bone area may simply reflect the association of inherently larger bony structures. However the beneficial associations demonstrated with cartilage morphology suggest that an increased knee height may confer a beneficial biomechanical environment to the chondrocyte of asymptomatic adults.

## Competing interests

The authors declare that they have no competing interests.

## Authors' contributions

AJT contributed to data analyses and manuscript writing AEW contributed to data acquisition, data analyses and manuscript drafting BJS contributed to data acquisition and manuscript drafting YW contributed to data acquisition and manuscript drafting PB contributed to data acquisition and manuscript drafting MDT contributed to data acquisition and manuscript drafting FMC contributed to data acquisition, data analyses and manuscript drafting. All authors read and approved the final manuscript.

## Pre-publication history

The pre-publication history for this paper can be accessed here:

http://www.biomedcentral.com/1471-2474/13/19/prepub
